# Effect of Fe and Si Content on Microstructure and Properties of Al-Cu-Li Alloys

**DOI:** 10.3390/ma19010147

**Published:** 2025-12-31

**Authors:** Tianyi Feng, Wei Zhao, Changlin Li, Ying Li, Xiwu Li, Zhicheng Liu, Lizhen Yan, Pengfei Xu, Hongwei Yan, Yongan Zhang, Zhihui Li, Baiqing Xiong

**Affiliations:** 1 State Key Laboratory of Nonferrous Structural Materials, China GRINM Group Co., Ltd., Beijing 100088, China; fty-micro@outlook.com (T.F.); lichanglin@grinm.com (C.L.); liying@grinm.com (Y.L.); yanlizhen@grinm.com (L.Y.); yanhongwei@grinm.com (H.Y.); zhangyongan@grinm.com (Y.Z.); lzh@grinm.com (Z.L.); xiongbq@grinm.com (B.X.); 2 GRIMAT Engineering Institute Co., Ltd., Beijing 101407, China; 3 General Research Institute for Nonferrous Metals, Beijing 100088, China; 4 Baotou Aluminum Co., Ltd., Baotou 014046, China; d20152186@163.com (Z.L.); lcl2186@126.com (P.X.); 5 School of Rare Earth Industry, Inner Mongolia University of Science and Technology, Baotou 014010, China

**Keywords:** Al-Cu-Li alloy, Fe and Si impurities, microstructure evolution, mechanical properties, fracture behavior

## Abstract

This study systematically investigates the effects of Fe and Si impurities on the microstructure and mechanical properties of Al-Cu-Li alloys. Five alloy compositions with controlled Fe (0.03–0.12 wt.%) and Si (0.03–0.12 wt.%) contents were fabricated and processed through homogenization, hot extrusion, solution treatment, and aging. Microstructural characterization demonstrates that Fe promotes the formation of coarse skeletal Al_7_Cu_2_Fe intermetallics, while Si facilitates the precipitation of blocky α-AlFeSi phases and eutectic Si particles. An elevated Fe content substantially deteriorates strength, ductility, and fracture toughness, primarily due to two mechanisms: the persistence of thermally stable impurity phases that serve as stress concentrators and preferential crack initiation sites throughout thermomechanical processing, and the consumption of Cu that reduces the volume fraction of primary T_1_ (Al_2_CuLi) strengthening precipitates. In contrast, Si exhibits comparatively moderate detrimental effects. The findings establish that stringent Fe control is essential for maintaining mechanical performance, whereas strategic Si adjustment offers a viable approach for cost management in recycled alloy production.

## 1. Introduction

Al-Li alloys are extensively utilized in the aerospace sector owing to their low density, high specific strength, and excellent cryogenic ductility. Third-generation Al-Li alloys, exemplified by the AA2195 alloy, achieve further performance enhancements through optimized compositional design, such as optimizing the Cu/Li ratio or adding microalloying elements. These refinements result in superior characteristics, including a reduced density, an enhanced strength–toughness balance, and diminished anisotropy. As a result, they are widely adopted in critical aerospace structures such as liquid oxygen tanks for heavy-lift launch vehicles and missile propellant tanks [1,2,3].

However, the escalating cost of Li and the inherent expense of Al additions substantially increase the raw material expenditure of Al-Li alloys, thereby constraining their application in complex structural components. In current research, utilizing recycled aluminum presents a viable pathway for cost reduction, but the associated impurity elements often compromise alloy performance [4,5,6,7]. Consequently, in order to enhance material utilization, minimizing production costs creates a critical need to elucidate the influence of impurity elements on the microstructure and properties of the Al-Cu-Li alloy.

During aluminum alloy processing, Fe and Si, which are the most common impurity elements, are difficult to remove entirely. These impurities originate not only from raw materials but may also be introduced during melting operations. These elements tend to form various insoluble or low-solubility intermetallic compounds with the matrix, significantly affecting microstructure, mechanical properties, and corrosion resistance. In demanding aerospace service environments, even trace impurities can induce severe performance degradation [8,9].

Yang et al. [10] observed in high-pressure die-cast Al-Si alloys that Fe primarily influences microstructure by forming acicular β-Al_5_FeSi phases and massive α-Al_15_(Fe, Mn)_3_Si_2_ phases. These phases, particularly the former, induce a stress concentration at the interface with the Al matrix under force loading, which significantly promotes strain localization, accelerates microcrack initiation, and drastically reduces the alloy’s strength and ductility. Chen et al. [11] observed that the presence of Fe and Si impurities during the friction stir additive manufacturing of 2195 aluminum–lithium alloy alters precipitation kinetics, thereby affecting the distribution and size of the T_1_ phase and leading to performance degradation. Ma et al. [12] investigated the effect of Fe content on fracture behavior in Al-Si-Cu casting alloys, finding that within the Fe content range of 0.2–1.0 wt.%, the impact energy of the alloy decreased significantly with increasing Fe content. This is attributed to the higher Fe content promoting the formation of coarse, lamellar β-Al_5_FeSi Fe-rich phases. These brittle intermetallic compounds readily serve as crack initiation sites, thereby degrading alloy performance. Xu et al. [13] investigated the evolution of the Fe-rich intermetallic phase in the 2219 Al-Cu alloy with different Fe contents (0.03, 0.10, 0.15, and 0.20 wt.%) during as-cast, homogenization, multi-directional forging and solution peak aging treatments, as well as its effects on mechanical properties and fracture behavior. The findings indicate that increasing Fe content significantly enhances both the quantity and size of the acicular Al_7_Cu_2_Fe phase, and that Fe content severely impacts the alloy’s elongation. Li et al. [14] investigated the effect of Fe content on the damage mechanism of the A319 aluminum alloy. The results show that in the low-Fe alloy (0.1 wt.% Fe), cracks initiate from the fracture of Si particles and Al_2_Cu phases in high stress concentration areas, and propagate along the eutectic Si and Al_2_Cu phases; while in the high-Fe alloy (0.8 wt.% Fe), cracks are mainly initiated by the fracture of acicular β-Al_5_FeSi intermetallic compounds and propagate along them. The increase in Fe content significantly increases the quantity and size of brittle Fe-based intermetallic compounds, making them the dominant damage path, thereby reducing the ductility and fracture toughness of the material.

Although existing studies have investigated the effects of impurity elements on various aluminum alloys, research specifically targeting Al-Cu-Li alloys remains relatively limited. Meanwhile, with the growing emphasis on the circular economy and the expanding application of recycled aluminum alloys, the controlled management of Fe and Si impurity levels has become a viable approach to cost reduction [15,16,17]. This study therefore aims to systematically elucidate the mechanisms by which Fe and Si impurities influence the microstructure and properties of Al-Cu-Li alloys. Specifically, it examines how Fe and Si contents affect the type, morphology, and distribution of impurity phases in as-cast microstructures and tracks their evolution during homogenization, hot deformation, and solution treatment. The findings will help establish impurity tolerance thresholds that do not compromise alloy performance, thereby providing key references for the industrial use of high-proportion recycled materials and low-cost raw and auxiliary inputs.

## 2. Materials and Methods

The experimental process involved ingot melting, homogenization heat treatment, solution treatment, hot extrusion, T8 aging treatment, microstructural analysis, and mechanical property testing.

The ingot melting process in this experiment utilized high-purity aluminum (99.99%), pure magnesium, pure copper, pure silver, metallic lithium, an aluminum–zirconium intermediate alloy (Al-5Zr), and an aluminum–titanium intermediate alloy (Al-10Ti) as primary raw materials. Impurity elements were precisely introduced by adding Al-20Fe and Al-20Si intermediate alloys. Five experimental alloy groups with varying Fe and Si contents were designed as follows: Alloy 1# (0.03 Fe, 0.03 Si), Alloy 2# (0.06 Fe, 0.03 Si), Alloy 3# (0.12 Fe, 0.03 Si), Alloy 4# (0.10 Fe, 0.09 Si), and Alloy 5# (0.03 Fe, 0.12 Si). The design rationale for these compositions is to first elucidate the primary influence trends of individual elements (via the 1#-3# series and the 1#-5# comparison) and to identify whether significant synergistic or antagonistic effects exist under dual high conditions. This approach provides clear direction for subsequent, more refined systematic experiments focused on interaction mechanisms. Melting was conducted in an electric resistance furnace at 710 °C, followed by casting into copper molds under an Ar atmosphere. The chemical compositions of the experimental alloys are shown in Table 1. Specimens were cut from the D/4 position of the five ingots.

All alloys in this experiment underwent identical heat treatment processes. The homogenization treatment regimen consisted of 460 °C/20 h + 505 °C/28 h. Hot extrusion at 460 °C produced 15 mm thick plates. The plates underwent solution treatment at 510 °C for 90 min, followed by water quenching at room temperature. After solution treatment, they were subjected to a 3.50% pre-stretch deformation. Subsequently, an aging treatment of 32 h at 155 °C was applied to the plates. The selection of aging temperature and pre-deformation level was based on a comprehensive analysis of precipitation behavior studies in Al-Li alloys [18,19,20,21,22,23,24,25,26].

Pre-stretch deformation was conducted using a WAW-1000 tester (Hualong, Shanghai, China) at a constant displacement rate of 1 mm/min. Room-temperature tensile testing was performed on a WDW-3100 electronic universal testing machine (Hualong, Shanghai, China) at a tensile rate of 2 mm/min, following GB/T 228.1-2010. Secondary phases and fracture morphology were observed using a JEOL JSM-7001F scanning electron microscope (JEOL Ltd., Tokyo, Japan) at 20 kV. SEM specimens underwent fine polishing without etching. Microstructural analysis was performed using a Talos F200 X G2 transmission electron microscope (TEM, Thermo Fisher Scientific, Waltham, MA, USA). TEM specimens were ground to 50 µm and subjected to dual-jet electrolytic polishing in a solution of 25% nitric acid and 75% methanol. The size and quantity of secondary phases and precipitates were statistically analyzed using ipp software(6.0). For each alloy state, we randomly selected no less than 5 representative fields of view from at least 3 independent metallographic specimens for analysis to ensure the universality of statistics and avoid regional deviation and a second independent measurement was carried out on 30% of the randomly selected images, confirming that the measurement method has good repeatability.

## 3. Results and Discussion

### 3.1. Microstructural Evolution

**Figure 1:**
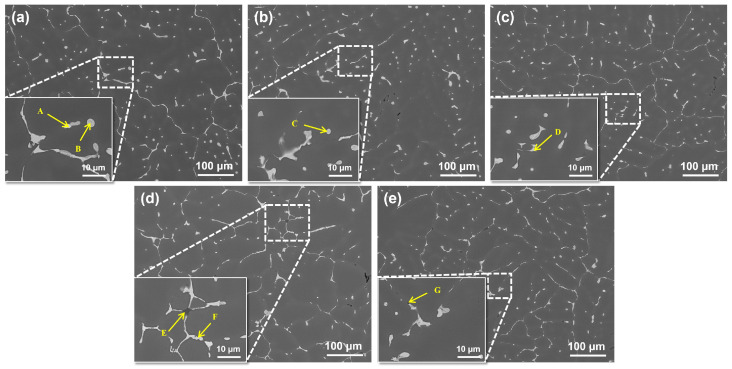
SEM images of as-cast alloys with different Fe and Si contents: (**a**) Alloy 1#; (**b**) Alloy 2#; (**c**) Alloy 3#; (**d**) Alloy 4#; (**e**) Alloy 5#.

Figure 1 shows the SEM microstructures of alloys with different Fe and Si contents in the as-cast state. From the backscattered electron images of the as-cast microstructure, the following phases are observed in the ingot: a gray-toned Al_2_Cu phase, grid-like AlCuMgAg phase, blocky Fe-rich phase, spherical Fe and Si phases, and black-toned Si particles.

Firstly, unreabsorbed Al_2_Cu and AlCuMgAg phases were observed in all alloys. Moving to the impurity-containing phases, distinct differences were noted among the alloys. In Alloy 1#, only occasional particles containing trace amounts of Fe and Si were found, which were sparse and contributed minimally to the total second phase area. In comparison, Alloy 2# contained a small but increased number of such Fe-Si-containing particles. A significant change occurred in Alloy 3#, which exhibited a markedly higher number density and notable coarsening of the Fe-rich phases. The microstructure became more complex in Alloy 4#, where Fe-Si intermetallics coexisted with discrete Si phases; notably, the Fe-Si intermetallics developed a distinct skeletal network morphology. Finally, under the condition of low Fe but high Si (Alloy 5#), the Si phase became dominant, appearing as discrete blocky or lamellar particles within the dendritic interstices. EDS analysis confirms that Fe primarily promotes the formation of Al-Cu-Fe-type impurity phases, whereas Si tends to precipitate as blocky or plate-like Si-rich phases. Under high-Fe conditions, Si participates in the formation of composite Al-Fe-Si intermetallics. The specific identities and compositional evolution of these phases require further analysis of the post-heat-treatment microstructure.

**Figure 2:**
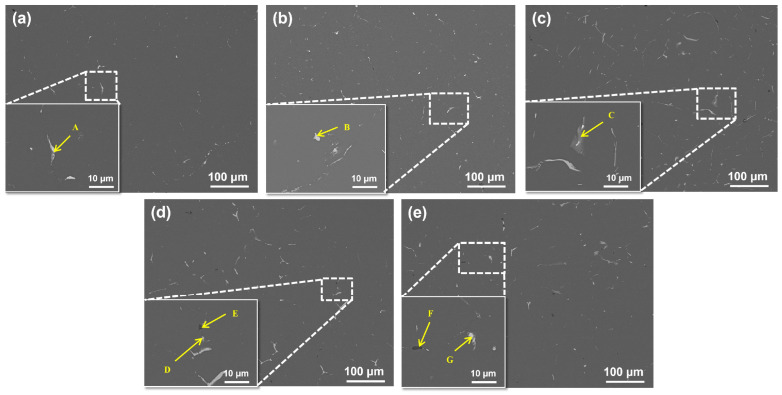
SEM images of homogenized alloys with different Fe and Si contents: (**a**) Alloy 1#; (**b**) Alloy 2#; (**c**) Alloy 3#; (**d**) Alloy 4#; (**e**) Alloy 5#.

Figure 2 shows the changes in the second phase of all the alloys after homogenization heat treatment. Following homogenization heat treatment, the second phase in the ingot primarily consists of Fe-rich and Si-rich phases. The SEM analysis of the homogenized alloys indicates that the non-equilibrium phases present in the as-cast condition, including the AlCuMgAg phase and Al_2_Cu phase, have been largely dissolved. However, Fe-rich phases persist in all alloys following homogenization. Alloy 1# contains significantly fewer secondary phases than the other compositions, with only isolated, fine Fe-rich particles remaining, resulting in the most uniform microstructure observed. This suggests the limited formation of Fe-rich intermetallics under low-Fe conditions. In Alloy 2#, the number of blocky Fe-rich phases increases, yet they remain resistant to dissolution. Alloy 3# displays a substantial rise in the population and size of Fe-rich phases, which exhibit elongated or skeletal morphologies and represent the most prominent secondary phases in this system. Alloy 4# contains both Fe-rich and Si-rich phases; the Si particles are primarily blocky or short rod-shaped, while the Fe-rich phases maintain an elongated morphology. The presence of Si facilitates the formation of Al-Fe-Si compounds, modifying the chemistry of some secondary phases. Alloy 5# also contains both phase types, though their size and quantity are reduced compared with Alloys 3# and 4#.

The EDS analysis results for the secondary phases across all alloys are summarized in Table 2. The data indicate that the atomic ratios within the Fe-rich and Si-rich phases remain consistent among the different alloys, suggesting uniform phase types. Based on comparisons from the literature, the most likely composition of this Fe-rich phase is Al_7_Cu_2_Fe, which primarily exhibits skeletal, coarse acicular, and blocky morphologies; this morphology and ternary composition are the hallmark features of the stable Al_7_Cu_2_Fe phase [27,28]. Although the absolute atomic ratio is affected by the matrix effect, a large amount of copper coexisting with iron can always be detected, and this feature distinguishes them from binary Al-Fe intermetallic compounds.

The Si-rich phase is identified as eutectic Si, characterized by its exceptionally high Si content and minimal levels of other elements, which is typical for silicon formed during terminal solidification. The Fe-Si composite phase can be observed to exhibit a blocky or short rod morphology, which is distinctly different from the typical acicular morphology of the detrimental β-Al_5_FeSi phase. In addition, EDS analysis shows that its Fe/Si atomic ratio is significantly greater than 1. Based on this speculation, combined with its morphology and composition, this phase is more likely to be the α-AlFeSi phase [29,30].

Overall, increasing Fe content promotes the growth in quantity and the coarsening of the grain size of Fe-rich secondary phases, whose morphology evolves from isolated particles to continuous skeletal or banded structures. The thermal stability of these phases consequently increases, leading to a significant reduction in their dissolution kinetics during subsequent homogenization treatment. In contrast, an increased Si content primarily promotes the formation of AlFeSi composite phases; under low-Fe conditions, additional Si tends to form independent Si-rich phases. Homogenization heat treatment effectively eliminates non-equilibrium Al_2_Cu and AlCuMgAg phases, though higher Fe and Si contents still leave substantial residual second phases.

**Figure 3:**
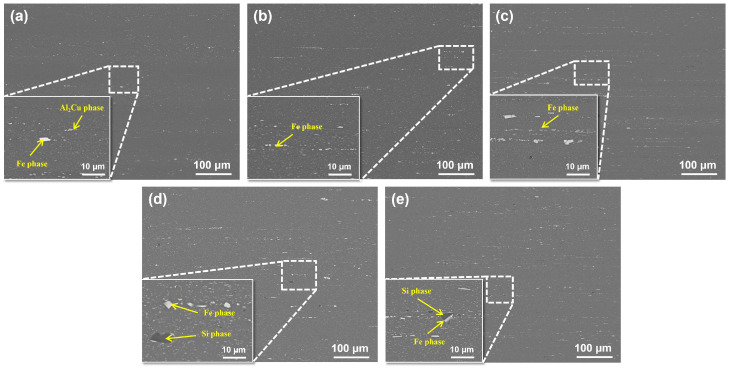
SEM images of squeezed alloys with different Fe and Si contents: (**a**) Alloy 1#; (**b**) Alloy 2#; (**c**) Alloy 3#; (**d**) Alloy 4#; (**e**) Alloy 5#.

Figure 3 presents the microstructures of the extruded alloys. The extrusion process induced substantial fragmentation of the secondary phases, particularly those containing Fe and Si. The resulting fragments are significantly refined and align in a chain-like distribution along the extrusion direction.

The EDS analysis results for the secondary phases across all alloys after homogenization are summarized in Table 3. Alloy 1# displays the least fragmentation, retaining only a minor amount of Fe-rich particles. With increasing Fe content, Alloys 2# and 3# show a greater number of deformed and fragmented Fe-rich phases, the size of which is more pronounced in Alloy 3#. Alloy 4# contains both fragmented Fe-rich and Si-rich phases; the former aggregate into continuous or semi-continuous bands, while the latter appear as blocky or flattened particles within these flow lines. In Alloy 5#, the Fe-rich phases are granular and the Si-rich phases, while relatively large, are limited in quantity, leading to a generally more dispersed distribution of secondary phases throughout the microstructure.

The above studies reveal that as Fe content increases, the quantity, size, and banded continuity of Fe phases in the extruded state are significantly enhanced, evolving from isolated particles to semi-continuous or continuous bands, thereby leading to increased thermal stability. Elevating Si content promotes the transformation of Fe into AlFeSi composite phases, altering their chemical composition and morphology. At lower Fe contents, independent Si-rich phases predominantly form, exhibiting a more isolated distribution.

**Figure 4:**
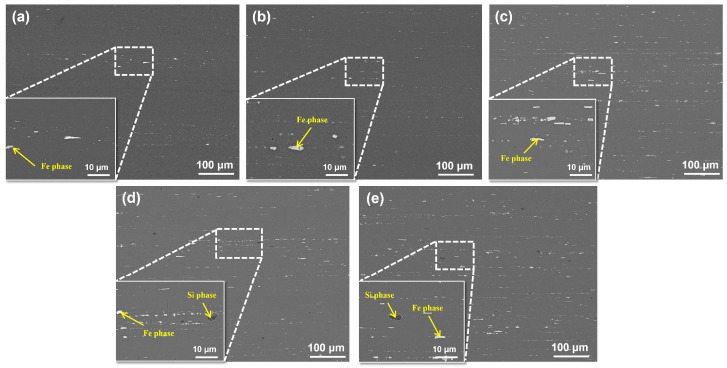
SEM images of solution treated alloys with different Fe and Si contents: (**a**) Alloy 1#; (**b**) Alloy 2#; (**c**) Alloy 3#; (**d**) Alloy 4#; (**e**) Alloy 5#.

Figure 4 displays microstructural images of all the alloys after solution treatment. Fe-rich and Si-rich phases remain observable within the structure, indicating that Fe and Si impurity phases remain insoluble at this temperature. After solution heat treatment, only a small amount of fine Fe-rich phase residues was observed in Alloy 1#, which exhibited isolated distributions and small sizes. In Alloys 2# and 3#, the Fe-rich phase coarsened with increasing Fe content. Both Alloy 4# and Alloy 5# exhibited the simultaneous presence of Fe-rich and Si-rich phases. However, Alloy 4# contained significantly higher quantities of both secondary phases, while Alloy 5# primarily showed the coexistence of the Si-rich phase with a minor amount of the Fe-rich phase. The solid solution microstructure observations confirm that both Fe-rich and Si-rich secondary phases are insoluble phases. The solid solution heat treatment failed to completely dissolve either phase, and the total quantity of the Fe-rich phase was substantially greater than that of the Si-rich phase.

**Figure 5:**
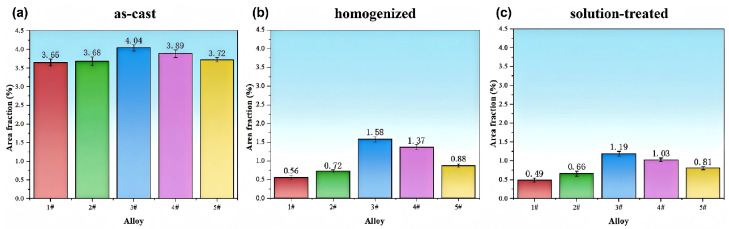
Statistical area fraction of the second phase in cast, homogenized, and solution-treated states of alloys with different Fe-Si contents: (**a**) as-cast; (**b**) homogenized; (**c**) solution-treated.

To further quantitatively analyze the effects of Fe and Si content on the second phase, we statistically analyzed the changes in the area fraction of the second phase during heat treatment for all alloys. The specific results are shown in Figure 5. The analysis of the data reveals that in the as-cast condition, the alloys contain a significant amount of secondary phases formed by non-equilibrium solidification, exhibiting relatively high initial area fractions: 3.65%, 3.68%, 4.04%, 3.89%, and 3.72%, respectively. At this stage, the secondary phase in the alloy microstructure primarily consists of Al_2_Cu and AlCuMgAg non-equilibrium eutectic phases formed during casting, along with insoluble phases containing Fe and Si. As the Fe content increases, the area fraction of the secondary phase in the alloy gradually increases, indicating that Fe significantly promotes the formation of the secondary phase. However, increasing the Si content has a limited effect on enhancing the area fraction of the secondary phase under low-Fe conditions.

Following homogenization heat treatment, the area fraction of the second phase in each composition decreased significantly. This was attributed to the dissolution of the soluble non-equilibrium Al_2_Cu phase and AlCuMgAg phase, as well as the weakening of some fine Fe-rich and Si-rich phases due to thermal diffusion. Alloy 1# exhibited the most pronounced decrease, from 3.65% to 0.56%, indicating that at low impurity levels, the alloy predominantly contains the soluble Al_2_Cu phase, resulting in a high homogenization efficiency. Alloy 3# retained the highest residual second phase area fraction, demonstrating that the Fe-rich phase exhibits significantly higher thermal stability than Al_2_Cu and is relatively difficult to dissolve back. Under high-Si conditions (Alloys 4# and 5#), the area fractions were 1.37% and 0.88%, respectively, with residual levels intermediate between high-Fe and low-Fe conditions. This reflects that the Si-rich phase also possesses stability during heat treatment but is generally more susceptible to re-dissolution compared with the Fe-rich phase.

The area fraction of the second phase in the solution treatment stage further decreased, but the rate of decrease was smaller compared with the homogenization stage. This indicates that the residual second phase in the alloy underwent fragmentation after extrusion. The finely fragmented chain-like second phase formed after fragmentation exhibited reduced re-dissolution during the solution treatment process, resulting in a higher residual ratio of coarse, poorly soluble Fe and Si phases. Among the alloys, Alloy 1# still exhibited the lowest residual of the second phase and the most uniform microstructure, while Alloy 3# maintained the highest content of the second phase.

### 3.2. Mechanical Properties

**Figure 6:**
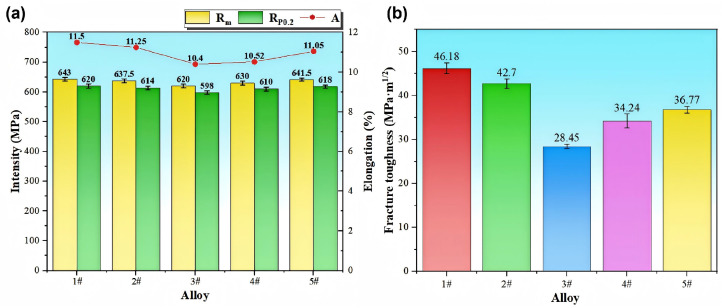
Columnar diagram of mechanical properties of alloys with different Fe-Si contents: (**a**) tensile property; (**b**) fracture toughness.

Figure 6 presents the tensile properties and fracture toughness of extruded strips with varying Fe and Si contents. In the experiment, for the tensile specimens of each group of alloys, five groups of parallel samples from the same position were selected for testing. Alloy 1#, with the lowest impurity content, demonstrates an optimal strength–ductility balance. A slight increase in Fe (Alloy 2#) leads to reductions in both strength and ductility. Alloy 3#, with the highest Fe content, exhibits the lowest strength and toughness among all compositions. While Alloy 4# exhibits a slight recovery in both strength and toughness after increasing its silicon content, Alloy 5# achieves strength levels comparable to Alloy 1# but with inferior elongation and toughness.

Comparative analysis confirms that the addition of Fe degrades the mechanical properties, resulting in decreases in strength, ductility, and toughness, with decreases of 3.5%, 7.8%, and 38.3%, respectively. In contrast, increasing the Si content has a weaker effect on strength, but also reduces ductility and toughness, with decreases of 0.2%, 3.9%, and 20.4%, respectively.

**Figure 7:**
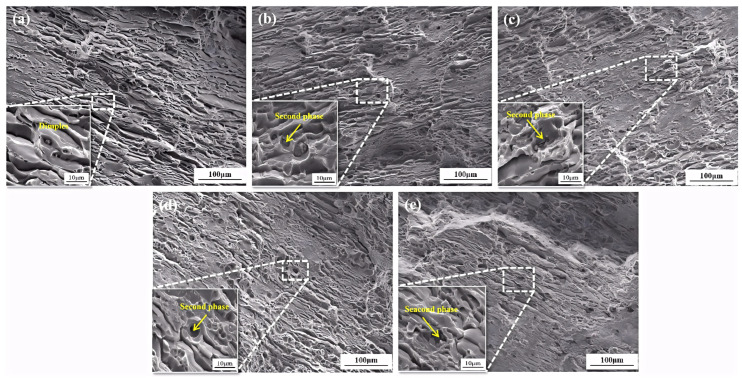
SEM images of tensile fracture of alloys with different iron and silicon contents: (**a**) Alloy 1#; (**b**) Alloy 2#; (**c**) Alloy 3#; (**d**) Alloy 4#; (**e**) Alloy 5#.

Figure 7 shows SEM observations of fracture surfaces from tensile specimens of alloys with varying Fe-Si content. An analysis of the fracture morphology reveals that all fracture surfaces exhibit distinct shear lips, tear ridges, and ductile pits, confirming that all alloys exhibit ductile fracture behavior. However, differences are observable in the microstructure at the fracture edges: In Alloy 1#, fine secondary phases are visible within the ductile pits, but no crack or microporosity aggregation is observed in these secondary phase regions. In Alloys 2# and 3#, with increasing Fe content, the size and quantity of the secondary phase within the ductile pits gradually increased. Additionally, distinct crack initiation was observed within the secondary phase, indicating that crack initiation and propagation are more likely to occur in the Fe-rich phase formed upon increased Fe content. Similarly, with increasing Si content in Alloys 4# and 5#, an increase in the second phase at the fracture surface compared with Alloy 1# can be observed. However, the occurrence of cracks within the second phase decreased. The performance degradation of Alloy 5# confirms that independently existing Si-rich phases (such as blocky α-AlFeSi or eutectic Si) are also detrimental to toughness. Although their morphology may be less sharp than that of skeletal Al_7_Cu_2_Fe, and the tendency for crack initiation is slightly lower, they are still effective damage sources.

### 3.3. Aging Precipitated Phase

**Figure 8:**
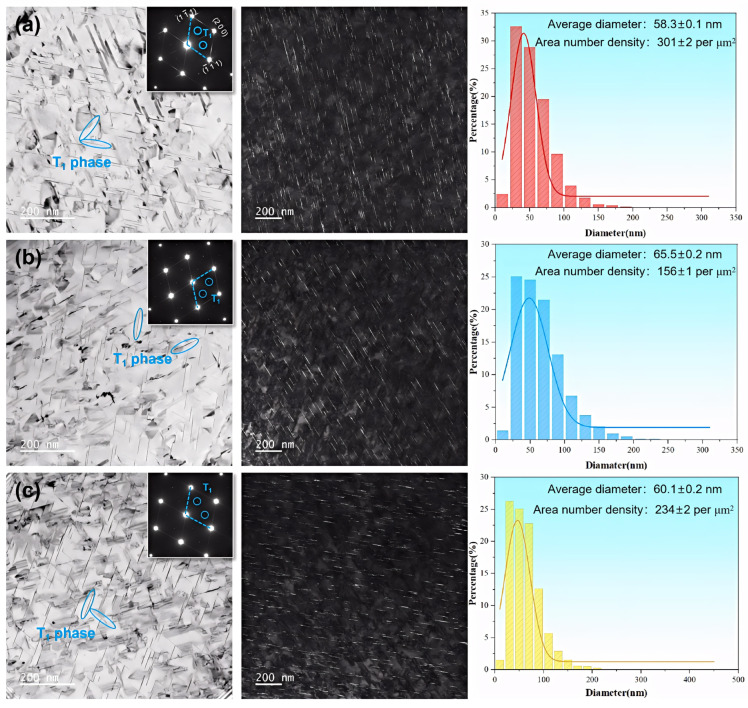
TEM images of alloys with different iron and silicon contents in the <110>_Al_ zone axis: (**a**) Alloy 1#; (**b**) Alloy 3#; (**c**) Alloy 5#.

This experiment selected Alloys 1#, 3#, and 5# to independently analyze the effects of Fe and Si elements on the aging precipitates. Figure 8 shows TEM images of the three groups of alloys along the <110>_Al_ axis zone, presenting the bright-field image, selected area electron diffraction (SAED) pattern, and dark-field image along the <110>_Al_ axis zone.

**Figure 9:**
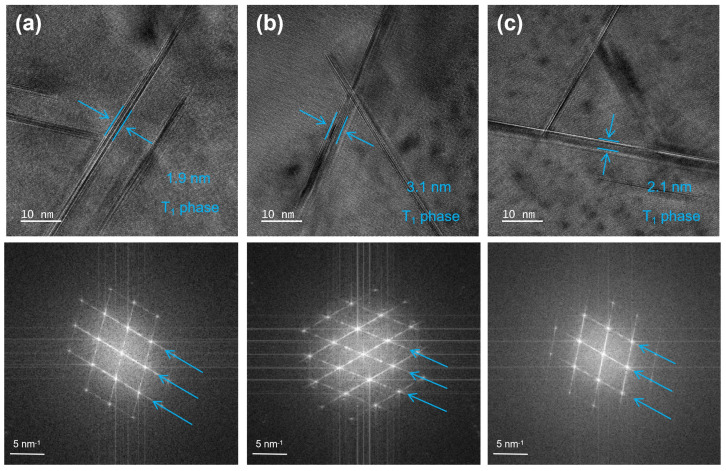
HRTEM and FFT analysis of T_1_ phase in alloys with different iron and silicon contents: (**a**) Alloy 1#; (**b**) Alloy 3#; (**c**) Alloy 5#.

The bright-field image in Figure 8 reveals high-density needle-like precipitates distributed along two directions, with an angle of approximately 109° between them. Further analysis using high-resolution TEM (HRTEM) and fast Fourier transform (FFT) in Figure 9 reveals that these precipitates correspond to the disk-shaped T_1_ phase, the primary strengthening phase in Al-Cu-Li alloys. This identification is confirmed by comparison with the literature and corresponds to the diffraction spots and blue fringes at the 1/3{220} and 2/3{220} positions in the SAED pattern [31,32].

To quantify the influence of Fe and Si content on precipitation characteristics, a statistical analysis of the average size and distribution of T_1_ (Al_2_CuLi) precipitates was conducted. In low-impurity Alloy 1#, the T_1_ phase exhibits the finest average diameter (58.3 nm) and a monomodal size distribution concentrated in the 45–60 nm range. Increasing the Fe content (Alloy 3#) results in a broader size distribution, a higher frequency of precipitates in the 50–100 nm range, and an increased average size of 65.5 nm. In contrast, elevating the Si content (Alloy 5#) yields an average T_1_ size of 60.1 nm with a primary distribution between 40 and 80 nm, indicating that Si addition has a less pronounced effect on coarsening compared with Fe.

The area fraction and thickness of T_1_ precipitates were statistically quantified from multiple micrographs. Alloy 1# exhibits the highest number density (301 μm^−2^), followed by Alloy 5# (234 μm^−2^), while Alloy 3# shows the lowest value (156 μm^−2^). Regarding precipitate thickness, Alloy 1# contains the thinnest T_1_ plates (~1.9 nm), followed by Alloy 5# (~2.1 nm), with Alloy 3# displaying the thickest precipitates (~3.1 nm). The marginal thickness difference between Alloys 1# and 5# contrasts with the significant coarsening observed in Alloy 3#.

**Figure 10:**
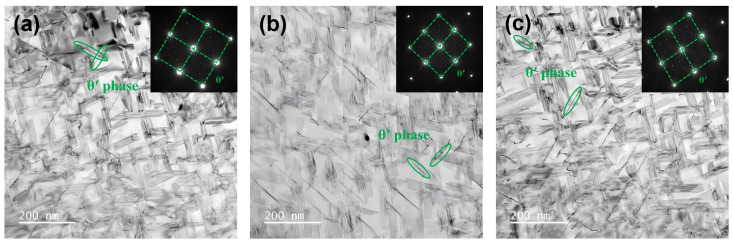
TEM images of alloys with different iron-silicon content in the <100>_Al_ zone axis: (**a**) Alloy 1#; (**b**) Alloy 3#; (**c**) Alloy 5#.

Due to the imaging constraints along the <110>_Al_ zone axis, only two of the four crystallographic variants of the precipitates growing on {111}_Al_ matrix planes are clearly resolved [33]. Consistently, Figure 10 confirms that a small fraction of disk-shaped θ′ (Al_2_Cu) phase is present in all three alloys [34,35]. In contrast to the T_1_ phase, the θ′ phase has three variants that form on {001}_Al_ planes, of which two mutually perpendicular variants are distinctly visible along the <100>_Al_ zone axis.

The strengthening contribution of precipitates in alloys is governed by multiple microstructural parameters, including diameter, thickness, and volume fraction. Elucidating the strengthening mechanisms of both T_1_ and θ′ phases is therefore essential for quantifying their individual contributions to overall strength. Since the main strengthening phase in Al-Cu-Li alloys is the T_1_ phase, and it can be found from the previous observations that the quantity of the θ′ phase in the alloy is less than that of the T_1_ phase, this study will focus on the strengthening contribution of the T_1_ phase for analysis [36,37]. Prior research has established that the strengthening efficacy of the T_1_ phase is size-dependent. Specifically, finer T_1_ precipitates (<100 nm) are more prone to dislocation shearing [18,35,38,39]. The average diameter of the T_1_ phase in this study falls within the size range generally recognized in the literature as being dominated by the shearing mechanism. Given the pronounced structural mismatch between the T_1_ phase and the aluminum matrix, this shearing process creates new interfaces with considerable coherency strain. To describe this interaction, Nie and Muddle [40] developed a precipitation strengthening model for the T_1_ phase, formulated as follows:

(1) $${∆\sigma }_{p}=\frac{1.211Md_{t}\gamma_{eff}^{\frac{3}{2}}}{t^{2}}\sqrt{\frac{bf_{V}}{\Gamma }}$$

In the model, M is the Taylor factor, d_t_ is the precipitate diameter, γ_eff_ is the interfacial energy of the T_1_ phase (0.107 J·m^−2^), t is the precipitate thickness, b is the magnitude of the Burgers vector (0.286 nm), f_V_ is the precipitate volume fraction, and Γ is the dislocation line tension, approximated as 0.5Gb^2^ (where G is the shear modulus, ~28 GPa). The volume fraction f_V_ is calculated as follows [19,36,39]:

(2) $$f_{V}=\frac{\pi N_{V}d_{t}^{2}t}{4}$$

In the equation, N_V_ denotes the number density of precipitates, d_t_ represents the calibrated diameter of precipitates, and t indicates the average precipitate thickness. The formula yields ${∆\sigma }_{p}\propto d_{t}^{2}N_{V}^{\frac{1}{2}}t^{-\frac{3}{2}}$, indicating that increases in both the diameter and number density of the precipitated phase enhance strength. Diameter exerts a greater influence, while increased thickness diminishes the strengthening contribution. Based on this, it can be concluded that the a alloy with high Fe content (Alloy 3#) has the lowest theoretical strength due to the largest average thickness of its precipitated phases.

Correlations among precipitate characteristics, fracture morphology, and tensile properties lead to the following conclusions. The addition of Fe will form a stable Al_7_Cu_2_Fe phase, which immobilizes a part of Cu and prevents it from participating in aging precipitation; at the same time, it will change the local solute distribution and precipitation environment, resulting in a decrease in the volume fraction of the main strengthening phase T_1_ and the coarsening of T_1_ phase. Concurrently, the brittle Fe-rich phases act as preferential sites for crack initiation and propagation. The synergistic effect of these two mechanisms—reduced precipitation strengthening and enhanced damage initiation—accounts for the marked deterioration in mechanical properties with increasing Fe content.

Although Si addition also degrades properties, its effect is less severe. Si interacts with Fe to form α-AlFeSi phases or discrete eutectic Si particles, both of which are less detrimental to mechanical properties than the Fe-rich intermetallics. Consequently, Si addition in high-Fe alloys mitigates the property loss. This study confirms that within the investigated composition range, Fe content is the dominant factor governing the properties of Al-Cu-Li alloys, while Si content exerts a secondary influence. The rational control of both elements enables cost reduction via recycled materials without incurring severe performance degradation.

## 4. Conclusions

This study systematically investigated the effects of varying Fe and Si impurity contents on the microstructure and properties of Al-Cu-Li alloys. The main conclusions are summarized as follows:

(1) Elevated Fe and Si contents promote the formation of impurity phases in Al-Cu-Li alloys. Fe-rich impurities predominantly manifest as skeletal Al_7_Cu_2_Fe intermetallics, while Si-containing phases primarily consist of blocky α-AlFeSi compounds and dark eutectic Si particles.
(2) An elevated Fe content substantially deteriorates the mechanical properties of the alloy, leading to significant reductions in both strength and ductility. In contrast, Si addition exerts a comparatively weaker influence.
(3) The degradation of alloy properties by impurities stems from two primary mechanisms. First, the skeletal morphology of Al_7_Cu_2_Fe phases induces stress concentration, serving as preferential sites for crack initiation and propagation during fracture. Second, Fe consumption of Al and Cu reduces the volume fraction of the primary strengthening T_1_ precipitates. The synergistic effect of these factors accounts for the overall performance deterioration.
(4) The impact of the moderate relaxation of Si content on performance is much smaller than that of Fe, which provides critical performance boundary data and a scientific feasibility basis for controlling raw material costs by adjusting the purity grade of raw materials (such as increasing the proportion of recycled materials) in specific application scenarios. At the same time, we will pay more attention to the combination of economic analysis and service requirements in subsequent research work.

## Figures and Tables

**Table 1:** Composition analysis results of Al-Cu-Li alloy (wt.%).

Alloy	Cu	Li	Mg	Zr	Ag	Si	Fe	Al
1#(0.03Fe, 0.03Si)	3.95	0.97	0.35	0.10	0.40	0.03	0.03	Bal.
2#(0.06Fe, 0.03Si)	3.97	0.98	0.38	0.09	0.40	0.03	0.06	Bal.
3#(0.12Fe, 0.03Si)	3.97	0.98	0.39	0.09	0.40	0.03	0.12	Bal.
4#(0.10Fe, 0.09Si)	3.97	1.04	0.34	0.11	0.41	0.09	0.10	Bal.
5#(0.03Fe, 0.12Si)	3.98	0.97	0.34	0.11	0.40	0.12	0.03	Bal.

**Table 2:** EDS analysis results of secondary phases in as-cast alloys with different Fe and Si contents (at. %).

Sample Number	Location Label	Al	Cu	Ag	Mg	Fe	Si	Phase
1#(0.03Fe,0.03Si)	A	62.97	32.81	-	4.23	-	-	Al_2_Cu phase
	B	70.01	22.01	0.65	7.32	-	-	AlCuMgAg phase
2#(0.06Fe,0.03Si)	C	71.33	23.13	0.34	5.19	-	-	AlCuMgAg phase
3#(0.12Fe,0.03Si)	D	77.00	6.47	-	-	16.53	-	Fe phase
4#(0.10Fe,0.09Si)	E	73.51	1.57	-	2.43	-	22.48	Si phase
	F	77.18	15.77	-	3.78	2.19	1.08	Al-Fe-Si phase
5#(0.03Fe,0.12Si)	G	56.38	0.43	-	1.68	-	41.51	Si phase

**Table 3:** EDS analysis results of secondary phases in homogenized alloys with different Fe and Si contents (at. %).

Sample Number	Location Label	Al	Cu	Mg	Fe	Si	Phase
1#(0.03Fe,0.03Si)	A	93.80	3.12	2.26	0.83	-	Fe phase
2#(0.06Fe,0.03Si)	B	80.01	13.85	-	6.13	-	Fe phase
3#(0.12Fe,0.03Si)	C	78.29	15.30	-	6.41	-	Fe phase
4#(0.10Fe,0.09Si)	D	77.22	15.31	3.78	5.57	1.90	Al-Fe-Si phase
	E	67.61	0.46	-	-	31.92	Si phase
5#(0.03Fe,0.12Si)	F	66.25	0.46	-	-	33.28	Si phase
	G	78.05	15.55	-	6.40	-	Fe phase

## Data Availability

The original contributions presented in this study are included in the article. Further inquiries can be directed to the corresponding authors.
